# Phosphatases Decrease Water and Urea Permeability in Rat Inner Medullary Collecting Ducts

**DOI:** 10.3390/ijms24076537

**Published:** 2023-03-31

**Authors:** Yanhua Wang, Janet D. Klein, Jeff M. Sands

**Affiliations:** Renal Division, Department of Medicine, Emory University, Atlanta, GA 30322, USA

**Keywords:** phosphatase, aldosterone, calcineurin, adrenomedullin, protein phosphatase 2A

## Abstract

We previously showed that the phosphatases PP1/PP2A and PP2B dephosphorylate the water channel, AQP2, suggesting their role in water reabsorption. In this study, we investigated whether protein phosphatase 2A (PP2A) and protein phosphatase 2B (PP2B or calcineurin), which are present in the inner medullary collecting duct (IMCD), are regulators of urea and water permeability. Inhibition of calcineurin by tacrolimus increased both basal and vasopressin-stimulated osmotic water permeability in perfused rat IMCDs. However, tacrolimus did not affect osmotic water permeability in the presence of aldosterone. Inhibition of PP2A by calyculin increased both basal and vasopressin-stimulated osmotic water permeability, and aldosterone reversed the increase by calyculin. Previous studies showed that adrenomedullin (ADM) activates PP2A and decreases osmotic water permeability. Inhibition of PP2A by calyculin prevented the ADM-induced decrease in water reabsorption. ADM reduced the phosphorylation of AQP2 at serine 269 (pSer269 AQP2). Urea is linked to water reabsorption by building up hyperosmolality in the inner medullary interstitium. Calyculin increased urea permeability and phosphorylated UT-A1. Our results indicate that phosphatases regulate water reabsorption. Aldosterone and adrenomedullin decrease urea or osmotic water permeability by acting through calcineurin and PP2A, respectively. PP2A may regulate water reabsorption by dephosphorylating pSer269, AQP2, and UT-A1.

## 1. Introduction

Vasopressin is the major hormone that increases water reabsorption in the kidney [1]. Vasopressin stimulation of osmotic water permeability is due, in large part, to stimulation of the aquaporin-2 (AQP2) water channel in the apical plasma membrane of collecting duct principal cells [2,3]. Vasopressin binds to type 2 vasopressin receptors (V2R) in the basolateral plasma membrane, resulting in stimulation of adenylyl cyclase, cyclic AMP (cAMP) production, and cAMP-dependent protein kinase A (PKA) activation. PKA promotes the phosphorylation of AQP2, thereby increasing its apical membrane accumulation and osmotic water permeability [2,3]. Control of water balance also involves the activity of the urea transporter UT-A1 in the inner medullary collecting duct (IMCD), which contributes to inner medullary interstitial hypertonicity [4]. Since vasopressin-induced activation of AQP2 and UT-A1 requires specific kinases to phosphorylate the transport proteins [5], it seems likely that reversing the effect of vasopressin to stimulate osmotic water reabsorption would require dephosphorylation of AQP2 and UT-A1 by specific phosphatases.

Previous studies indicate that aldosterone, a steroid hormone that is produced in the adrenal cortex, modulates electrolyte and water homeostasis in the distal tubule through a genomic mechanism by which the conjugate of aldosterone and its intracellular mineralocorticoid receptors (MRs) translocates into the nucleus and regulates gene transcription of the epithelial sodium channel (ENaC) and the signaling proteins and kinases that mediate ENaC activity, such as serum/glucocorticoid kinases (SGKs) [6,7,8]. In addition to its genomic effects, aldosterone has rapid actions that are independent of transcription and translation. Aldosterone activates protein phosphatase PP2B (calcineurin) in rat cortical collecting ducts through an MR-dependent but transcription-independent mechanism [9,10]. We showed that inhibiting calcineurin alters the phosphorylation and the activity of UT-A1 and AQP2 in the IMCD [11,12] and that aldosterone directly decreases vasopressin-stimulated osmotic water and urea reabsorption [13]. These findings support the hypothesis that aldosterone regulates vasopressin-stimulated osmotic water and urea reabsorption through a calcineurin-dependent mechanism.

In addition to calcineurin, other phosphatases may also be involved in the regulation of water and urea transport. Proteomic analyses show that protein phosphatases (PP) PP1, PP2A, and PP2B interact with AQP2 [14] and that PP2A interacts with UT-A1 [15]. We previously showed that PP1/PP2A regulate the phosphorylation of AQP2 in rat IMCDs [12]. Adrenomedullin (ADM), a vasodilator peptide produced in various tissues including the kidney, activates PP2A in mesangial cells [16] and hypothalamic neurons [17]. ADM inhibits osmotic water permeability in rat IMCDs [18]. These findings suggest that, in addition to calcineurin, PP2A may also regulate water homeostasis through changes in AQP2 and UT-A1 phosphorylation. An ADM-induced decrease in water reabsorption may be mediated by an increase in PP2A activity.

The present study provides data that extends our understanding of the effects of phosphatases on osmotic water and urea permeability, which highlights a new direction for therapies for diseases related to water imbalance. We first investigated whether calcineurin is involved in water reabsorption. Next, we examined the role of PP2A in regulating AQP2 and UT-A1. Finally, the effects of aldosterone and ADM on osmotic water and urea permeability were tested. The primary rationale for this aspect of our study is that the two hormones activate the two phosphatases, calcineurin and PP2A, respectively. We found that: (1) PP2A and PP2B regulate osmotic water and urea permeability in rat IMCDs; (2) aldosterone and ADM inhibit osmotic water permeability through activation of PP2B and PP2A, respectively.

## 2. Results

### 2.1. Inhibition of Calcineurin Increased Urea and Osmotic Water Permeability

**Figure 1:**
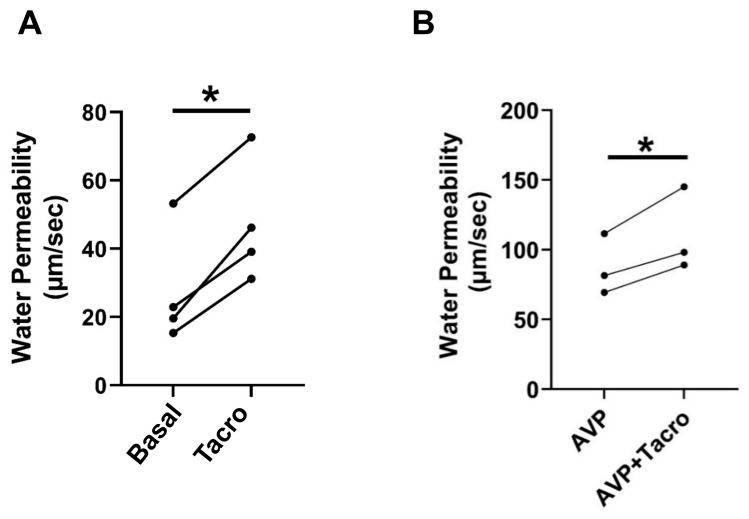
Inhibition of calcineurin by tacrolimus increased osmotic water permeability. (**A**) Terminal IMCDs were perfused with 800 nM tacrolimus (Tacro) for 30 min. Each line represents a separate IMCD from a different rat. * = *p* < 0.05 basal vs. tacrolimus, *n* = 4 rats/condition. (**B**) Tacrolimus (30 min) increases vasopressin (AVP, 50 pM)-stimulated water permeability in rat IMCDs. * = *p* < 0.05 AVP vs. AVP + tacrolimus, *n* = 3 rats/condition.

We previously showed that inhibition of calcineurin by its inhibitor, tacrolimus, significantly increases urea permeability in rat IMCDs [4]. In this study, to test whether calcineurin regulates osmotic water permeability, rat terminal IMCDs were perfused first without and then with tacrolimus in the bath. Osmotic water permeability was evaluated by measuring the raffinose (a volume marker) content in collected fluids from the IMCD lumen. Our results showed that inhibition of calcineurin by tacrolimus increased osmotic water permeability in perfused rat IMCDs from 28 ± 9 to 47 ± 9 µm/s (*n* = 4, *p* < 0.05, Figure 1A).

### 2.2. Inhibition of Calcineurin Increased Vasopressin-Stimulated Osmotic Water Permeability

Vasopressin is a major hormone that regulates osmotic water permeability. To test whether calcineurin modulates vasopressin-stimulated osmotic water permeability, rat terminal IMCDs were perfused with vasopressin, followed by tacrolimus in the bath. Tacrolimus increased vasopressin-stimulated osmotic water permeability from 87 ± 13 to 111 ± 17 µm/s (*n* = 3, *p* < 0.05, Figure 1B).

### 2.3. Inhibition of Calcineurin Did Not Change Aldosterone-Reduced Osmotic Water Permeability

**Figure 2:**
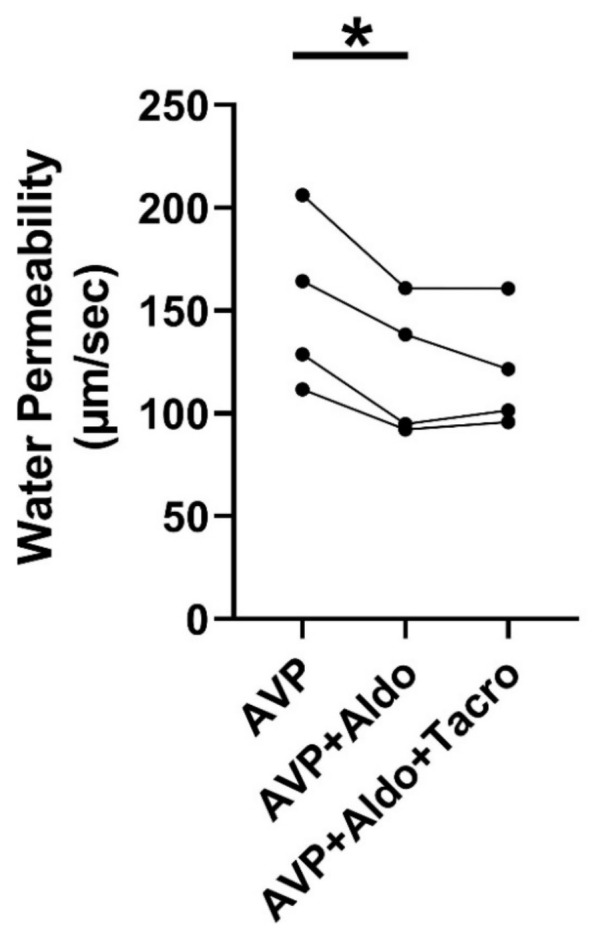
Inhibition of calcineurin did not change aldosterone-reduced osmotic water permeability. Terminal IMCDs were perfused with 50 pM vasopressin (AVP) for 15 min, subsequently with 500 nM aldosterone (Aldo) for 30 min, and finally with 800 nM tacrolimus (Tacro). * = *p* < 0.05 AVP vs. AVP + Aldo, *n* = 4 rats/condition. Each line represents a separate IMCD from a different rat.

We previously indicated that aldosterone inhibits vasopressin-stimulated osmotic water permeability [13]. To test whether aldosterone reduces vasopressin-regulated water reabsorption through calcineurin, rat terminal IMCDs were first perfused with vasopressin, followed by aldosterone and then tacrolimus, which were added to the bath in the presence of vasopressin. Aldosterone reduced vasopressin-stimulated osmotic water permeability from 153 ± 21 to 122 ± 17 µm/s (*n* = 4, *p* < 0.05, Figure 2). The subsequent addition of tacrolimus to the bath did not change aldosterone-inhibited osmotic water permeability (Figure 2).

### 2.4. Inhibition of PP2A Increased Osmotic Water Permeability

**Figure 3:**
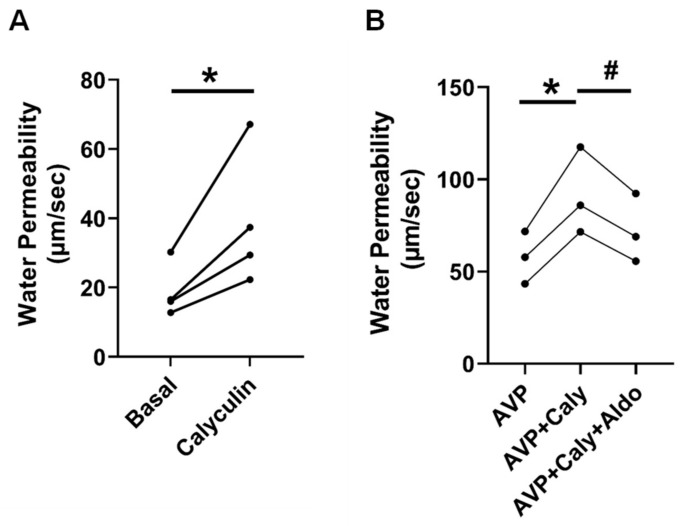
Inhibition of PP2A by calyculin increased osmotic water permeability. (**A**) Terminal IMCDs were perfused with 35 nM calyculin for 30 min. * = *p* < 0.05 basal vs. calyculin, *n* = 4 rats/condition. (**B**) Inhibition of PP2A by calyculin (Caly) increased AVP (vasopressin)-stimulated osmotic water permeability. Terminal IMCDs were perfused with 50 pM vasopressin (AVP) for 15 min, subsequently with 35 nM Caly, and finally with 500 nM aldosterone (Aldo). * = *p* < 0.05 AVP vs. AVP + Caly, # = *p* < 0.05 AVP + Caly vs. AVP + Caly + Aldo, *n* = 3 rats/condition. Each line represents a separate IMCD from a different rat.

Previous studies imply that PP2A is involved in the regulation of osmotic water permeability [14]. To verify whether PP2A regulates osmotic water permeability, rat terminal IMCDs were perfused first without and then with calyculin, a PP2A inhibitor, in the bath. Inhibition of PP2A by calyculin increased osmotic water permeability from 19 ± 4 to 39 ± 10 µm/s (*n* = 4, *p* = 0.03, Figure 3A).

To test whether PP2A regulates vasopressin-stimulated osmotic water permeability, rat terminal IMCDs were perfused with vasopressin, subsequently with calyculin, and finally with aldosterone in the bath. Inhibition of PP2A by calyculin increased vasopressin-stimulated osmotic water permeability from 58 ± 8 to 92 ± 14 µm/s (*p* < 0.05). The subsequent addition of aldosterone reversed the calyculin-induced osmotic water permeability from 92 ± 14 to 72 ± 11 (*n* = 3, *p* < 0.05, Figure 3B).

### 2.5. Inhibition of PP2A Increased Urea Permeability and UT-A1 Phosphorylation

**Figure 4:**
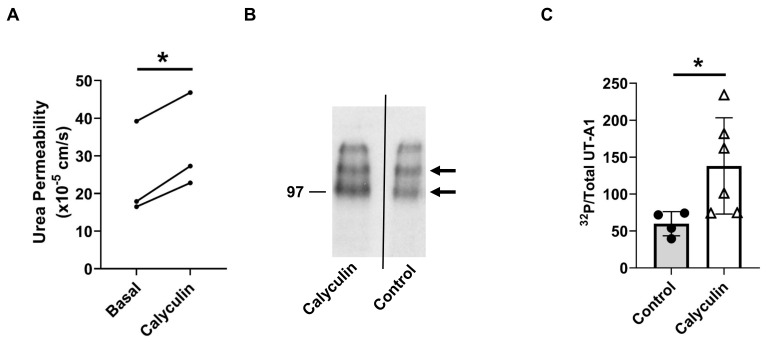
Inhibition of PP2A by calyculin increased urea permeability. (**A**) Terminal IMCDs were perfused with 35 nM calyculin for 30 min. Each line represents a separate IMCD from a different rat. * = *p* < 0.05 basal vs. calyculin, *n* = 3 rats/condition. (**B**) Calyculin increased phosphorylated UT-A1. SDS-polyacrylamide gels of the immunoprecipitated UT-A1 from kidney inner medulla of control and calyculin-treated rats. Inner medullas were radiolabeled with [^32^P], UT-A1 was immunoprecipitated from equal amounts of IM lysate. Precipitated UT-A1 was separated by SDS-polyacrylamide gel electrophoresis and the dried gels were subjected to autoradiographic analysis. Arrows denote the molecular weights of proteins (UT-A1: 97–117 kDa). (**C**) Bars = average band density ± s.e., *n* = 4–6, * = *p* < 0.05.

Previous studies suggest that PP2A may regulate urea permeability [15]. To confirm whether PP2A regulates urea permeability, rat terminal IMCDs were perfused with calyculin in the bath. Inhibition of PP2A by calyculin increased urea permeability from 25 ± 7 to 32 ± 7 × 10^−5^ cm/s (*n* = 3, *p* = 0.01, Figure 4A). To test the role of PP2A on UT-A1 phosphorylation, rat inner medullary (IM) tissues were incubated with calyculin for 30 min. Tissue lysates were analyzed by autoradiography, probing for phosphorylated UT-A1. Inhibition of PP2A by calyculin increased the abundance of phosphorylated/total UT-A1 (control: 60 ± 8; calyculin-treated: 138 ± 27, *n* = 6, *p* < 0.05, Figure 4B,C).

### 2.6. Inhibition of PP2A Prevented the Decrease in Osmotic Water Permeability by ADM

**Figure 5:**
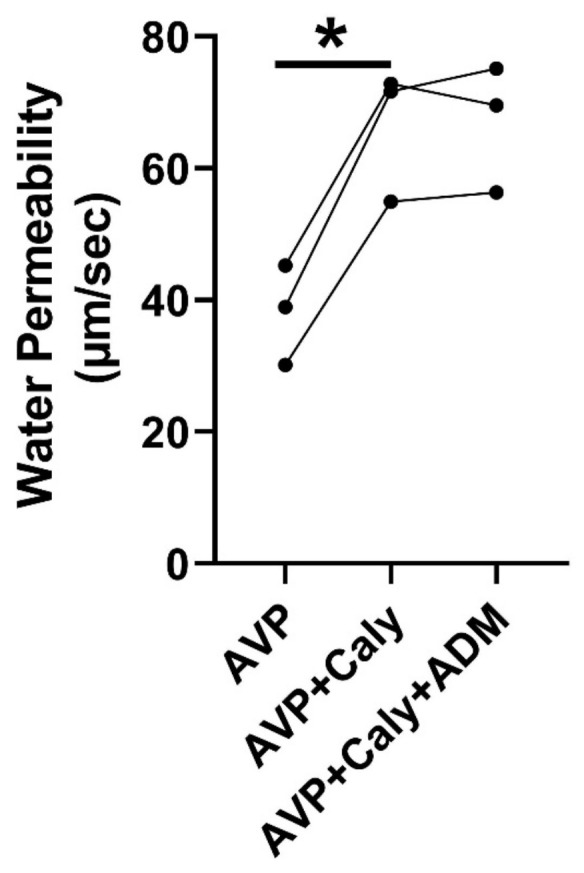
Inhibition of PP2A by calyculin (Caly) prevented ADM (adrenomedullin)-inhibited osmotic water permeability. Terminal IMCDs were perfused with 50 pM vasopressin (AVP), subsequently with 35 nM Caly, and finally with 100 nM ADM. * = *p* < 0.05 AVP vs. AVP + Caly, *n* = 3 rats/condition. Each line represents a separate IMCD from a different rat.

ADM reduces osmotic water permeability in rat IMCDs [18] and activates PP2A in mesangial cells [16] and hypothalamic neurons [17]. To test whether PP2A is involved in ADM-reduced osmotic water permeability, rat terminal IMCDs were perfused with vasopressin, subsequently with calyculin, and finally with ADM in the bath. Inhibition of PP2A by calyculin increased vasopressin-stimulated osmotic water permeability from 38 ± 4 to 66 ± 6 µm/s (*p* < 0.05). The subsequent addition of ADM did not change the osmotic water permeability (Figure 5).

### 2.7. ADM Mediates Phosphorylation of AQP2

**Figure 6:**
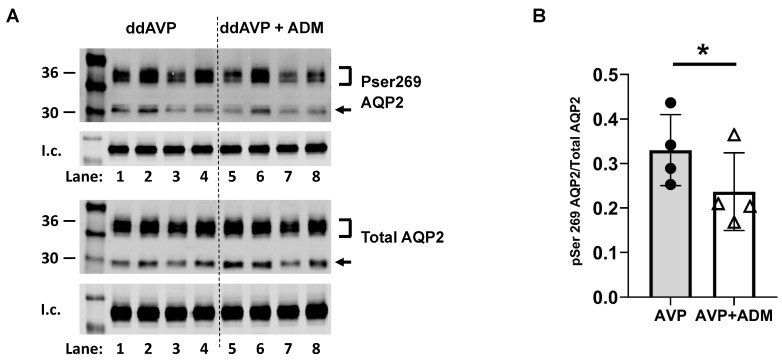
ADM decreased the phosphorylation of AQP2 at serine 269. (**A**) Western analysis of kidney inner medullary (IM) lysate from ddAVP (1-deamino-8-D-arginine vasopressin, AVP) and ddAVP + ADM (adrenomedullin)-treated IM probed for total (bottom) and pSer269 (top) AQP2. Brackets indicate the glycosylated AQP2 protein between 35 and 45 kDa, and the arrow indicates the un-glycosylated AQP2 protein at 29 kDa. The tubulin loading control (l.c.) is shown beneath each blot. The two kidney IMs from the same animal were randomly assigned to the AVP or the AVP + ADM groups. The matched pair comparisons were achieved by comparing Lane 1 with Lane 5; Lane 2 with Lane 6; Lane 3 with Lane 7; and Lane 4 with Lane 8. (**B**) Bar graph showing the pSer269AQP2/total AQP2 density ratio. Bars: Mean ± s.e., *n* = 4, * = *p* < 0.05 AVP vs. AVP + ADM.

Previous experiments show that ADM inhibits the phosphorylation of AQP2 at serine 256 and phosphorylates AQP2 at serine 261 [18]. However, no evidence has been collected on the phosphorylation of AQP2 at serine 269. Phosphorylation of AQP2 at serine 269 is stimulated by AVP and plays a critical role in water reabsorption [19,20]. To test the role of ADM in vasopressin-stimulated AQP2 phosphorylation at serine 269, IM tissues were incubated in an isotonic medium with vasopressin for 20 min and then treated with ADM for 30 min. Tissue lysates were analyzed by western blot, probing for total AQP2 and pSer269 AQP2 (Figure 6A). ADM significantly decreased pSer269 AQP2 by 27% (AVP: 0.33 ± 0.04; AVP + ADM-treated: 0.24 ± 0.04; *n* = 4, *p* < 0.05; Figure 6B). We probed for AQP2 phosphorylation at serine 269 in tissues without any AVP treatment, and phosphorylated serine 269 was not detected. 

## 3. Discussion

The primary finding of the present study is that two phosphatases, calcineurin (PP2B) and PP2A, are involved in vasopressin-stimulated water and urea reabsorption. Previous studies show that calcineurin and PP2A regulate the phosphorylation of AQP2 and its membrane accumulation in the rat inner medulla [12]. However, there are no previous studies that provide direct functional evidence confirming the role of phosphatases in water reabsorption. Therefore, we tested for functional effects using isolated, perfused rat IMCDs. Urea transport contributes to medullary hypertonicity and is inextricably linked to water movement [4,15]. Therefore, understanding how phosphatases affect UT-A1 is also important for comprehending its role in water balance. In this study, we used two phosphatase inhibitors (tacrolimus and calyculin) as well as two hormones (aldosterone and adrenomedullin) to investigate the role of specific phosphatases in urea and osmotic water permeability. Aldosterone and adrenomedullin activate calcineurin and PP2A, respectively. In vivo, the levels of both aldosterone [21,22] and adrenomedullin [23] increase in response to vasopressin stimulation. Given that both ADM and aldosterone regulate osmotic water reabsorption and stimulate phosphatases, the two hormones are the appropriate hormones that allow us to explore the underlying mechanisms for the role of phosphatases in vasopressin-stimulated osmotic water reabsorption.

Our published data indicate that aldosterone rapidly decreases vasopressin-stimulated osmotic water permeability in rat terminal IMCDs [13], suggesting its ability to reduce water retention caused by high levels of vasopressin. However, the underlying mechanism for the acute inhibition of water reabsorption by aldosterone is unclear. As a steroid hormone, aldosterone modulates sodium channel and sodium transporter activities mainly through a genomic mechanism. Aldosterone binds to MRs, thereby controlling the transcription of the sodium channel and transporter genes [6,7]. The product of these genes eventually alters the activity of ionic transport systems located in the apical and basolateral plasma membranes of epithelial cells. In addition, aldosterone can mediate channel and transporter activities through a non-genomic mechanism [24,25,26]. Calcineurin appears to be such a mechanism, allowing for the rapid effect of aldosterone on vasopressin-stimulated osmotic water reabsorption since it is activated by aldosterone independent of transcription and translation [9,10].

Tacrolimus inhibits calcineurin by acting on calcium-dependent biological processes including immune responses, signal transduction, and muscle development [27]. We first tested whether inhibition of calcineurin by tacrolimus changes osmotic water permeability. Our results indicate that tacrolimus increases both basal and vasopressin-stimulated osmotic water permeability (Figure 1), suggesting that calcineurin is involved in the regulation of water reabsorption. To confirm the role of calcineurin in aldosterone-regulated osmotic water reabsorption, we perfused rat IMCDs with aldosterone, followed by inhibition of calcineurin with tacrolimus in the presence of vasopressin. If calcineurin is downstream of aldosterone, it should be activated, leading to dephosphorylation of AQP2 and increasing water excretion. Our results show that aldosterone reduced vasopressin-stimulated osmotic water permeability, and the decrease in water permeability caused by aldosterone was not changed by the further addition of tacrolimus (Figure 2). This suggests that aldosterone’s activation of calcineurin maximally dephosphorylated AQP2 and prevented its inhibition by tacrolimus.

In addition to calcineurin, PP2A is likely to be involved in regulating AQP2 [14] and UT-A1 [15]. Our results show that PP2A inhibition by calyculin [28,29] increased UT-A1 phosphorylation (Figure 4B,C). Data from isolated perfused tubules show that calyculin significantly increased urea permeability (Figure 4A) and osmotic water permeability (Figure 3) in rat IMCDs. The increase in urea permeability is consistent with the increase in UT-A1 phosphorylation (Figure 4). The elevated osmotic water reabsorption (Figure 3) is in line with our previous finding that calyculin significantly increases the abundance of pS256-AQP2 [12]. These findings suggest that PP2A decreases osmotic water permeability and urea permeability by dephosphorylating AQP2 and UT-A1, respectively. Aldosterone reversed the increase in osmotic water permeability by calyculin (Figure 3B), suggesting that in addition to aldosterone-activated calcineurin, PP2A is a supplemental mechanism that contributes to water homeostasis.

Previous studies show that ADM is a vasodilator peptide that stimulates PP2A [16,17]. ADM decreases osmotic water permeability, which is a cAMP-independent process [18]. These findings imply a role for PP2A in ADM-inhibited osmotic water permeability. Our results demonstrate that calyculin increases vasopressin-stimulated osmotic water permeability. The increase in osmotic water permeability by calyculin was not changed by ADM (Figure 5), suggesting that the pre-inhibition of PP2A by calyculin prevents the decrease in osmotic water permeability by ADM. Protein analysis showed that ADM decreased AQP2 phosphorylation at serine 269 (Figure 6), suggesting that serine 269 may be a target site where PP2A dephosphorylates AQP2. We also found that phosphorylated serine 269 was not detectable in tissues without vasopressin stimulation, which supports the concept that phosphorylation at serine 269 is a vasopressin-dependent event [20]. Therefore, we could not use tissue that was not treated with vasopressin to determine whether serine 269 is a phosphorylation site for PP2A. In combination with our previous findings that ADM decreases AQP2 phosphorylation at serine 256 and increases AQP2 phosphorylation at serine 261 [18], further studies are required to determine whether ADM-induced AQP2 phosphorylation at these amino acid residues is mediated by PP2A.

## 4. Materials and Methods

### 4.1. Animals

All animal surgical protocols and procedures were approved by the Emory Institutional Animal Care and Use Committee (protocol number “PROTO 201800110”) and adhere to the NIH standards for animal use. These studies used both male and female rats from Charles River Laboratories, Wilmington, MA, USA. To measure osmotic water permeability (Pf) and urea permeability, rats weighing 50–75 g were sacrificed by decapitation to avoid any anesthesia effect, and the kidneys were quickly dissected to remove the IM. The rats used in this study were 3–4 weeks old. Urine concentrating ability is fully developed at that age, and the tubules are in good condition for microdissection. To test protein phosphorylation, rats weighing 100–140 g were sacrificed, and IMs were dissected from the kidneys and placed on ice until tissue treatments.

### 4.2. Tubule Perfusion

IMs were transferred to a dissection dish, and terminal IMCDs were microdissected in a dissection buffer at 17 °C. The dissecting solution contained (in mM): 125 NaCl, 25 NaHCO_3_, 2 CaCl_2_, 2.5 K_2_HPO_4_, 1.2 MgSO_4_, and 5.5 glucose. The solution osmolality was adjusted to 430 mosmol/kg H_2_O with NaCl only for the purpose of dissecting tubules [30]. The perfusion and bath solutions were identical to the dissection medium, except that 5 mM raffinose was added to both the perfusate and the bath, and an additional 70 mM NaCl was added to the bath when measuring osmotic water permeability to create a bath-to-lumen osmolality gradient of ~140 mosmol/kg H_2_O [1]. When measuring urea permeability, 5 mM raffinose was added to the perfusate, and 5 mM urea was added to the bath. All solutions were gassed continuously with 95% air and 5% CO_2_ before and during the dissection and perfusion [1].

Single IMCDs were dissected, mounted on glass pipettes, and perfused as described [1]. In general, 45 min after warming the tubules to 37 °C in 2 mL of bath solution, two initial collections of 2–3 min at 6–8 nL/min were made. Treatments were added to the bath, and tubules were allowed to equilibrate for 30 min, then two further 2–3 min collections were made. To measure osmotic water permeability, collected solutions were assayed for raffinose content by ultramicrofluorometry [31]. Raffinose was used as a volume marker. To measure urea permeability, collected solutions were assayed for urea content by ultramicrofluorometry [1,32]. Osmotic water and urea permeabilities were calculated as described [1]. Biological variation in baseline osmotic water or urea permeability between different animals has been recognized for many years [33]. Therefore, we used the baseline value of each tubule as its own control.

To assess the contribution of calcineurin to osmotic water permeability, it was measured under the basal (no vasopressin) condition, then tacrolimus (800 nM) (MilliporeSigma, Burlington, MA, USA), a calcineurin inhibitor, was added to the bath for 30 min, after which 2 collections were made for raffinose determination. To determine whether calcineurin regulates vasopressin-stimulated osmotic water permeability, vasopressin (50 pM) (MilliporeSigma, Burlington, MA, USA) was added to the bath and osmotic water permeability was measured, then tacrolimus (800 nM) was added to the bath and osmotic water permeability was measured again. To determine whether aldosterone regulates vasopressin-stimulated osmotic water permeability through calcineurin, vasopressin-stimulated osmotic water permeability was measured. Then, aldosterone (500 nM) (MilliporeSigma, Burlington, MA, USA) was added to the bath to activate calcineurin, and osmotic water permeability was measured. Thirty minutes after the addition of aldosterone, tacrolimus (800 nM) was added to the bath for a further 30 min, and osmotic water permeability was measured.

To determine whether PP2A is involved in regulating osmotic water or urea permeability, basal osmotic water or urea permeability was measured, and then calyculin (35 nM) (MilliporeSigma, Burlington, MA, USA) was added to the bath for 30 min to inhibit PP2A. Then 2 further collections were made for raffinose or urea determination. To determine whether PP2A regulates vasopressin-stimulated osmotic water permeability, vasopressin (50 pM) was added to the bath, and osmotic water permeability was measured. Next, calyculin (35 nM) was added to the bath, and osmotic water permeability was measured. A total of 30 min after the addition of calyculin, aldosterone (500 nM) was added to the bath for a further 30 min, and osmotic water permeability was measured. To determine whether ADM inhibits osmotic water permeability by activating PP2A, tubules were perfused with vasopressin (50 pM) in the bath. Next, calyculin (35 nM) was added to the bath. Thirty minutes after the addition of calyculin, ADM (100 nM) (Abcepta, San Diego, CA, USA) was added to the bath for a further 30 min. The osmotic water permeability was measured at each period of perfusion.

### 4.3. Tissue Incubation

To measure AQP2 phosphorylation, one of the two IMs from the same animal was assigned as a control, and the other IM was assigned as a treatment. IMs were sectioned into ~1 mm^3^ tissue pieces. The dissecting HBSS solution contained (in mg/L): 400 KCl, 60 KH_2_PO_4_; 350 NaHCO_3_; 8000 NaCl; 90 Na_2_HPO_4_·7H_2_O; and 1000 dextrose anhydrous. The IM pieces were first incubated at 37 °C for 10 min in isotonic Hanks’ balanced salt solution (HBSS) to establish baseline resting conditions and then stimulated by 50 pM vasopressin in both the control and treatment groups. To test the effect of ADM, the treatment group was stimulated at 37 °C for 30 min with 100 nM ADM. The reactions were terminated by placing the samples on ice and replacing the incubation solutions with an ice-cold homogenization buffer (10 mM triethanolamine, 250 mM sucrose, and 10% sodium dodecyl sulfate). Tissues were homogenized and analyzed for AQP2 phosphorylation by Western blot. The AQP2 phosphorylation level was calculated as the ratio of phosphoprotein/total protein to determine if the phosphorylation level per protein changes.

### 4.4. Western Blot Analysis

Samples of whole IM protein lysate (20 µg/lane), one animal per lane, were size separated by SDS-PAGE on 12.5% gels and then electroblotted to polyvinylidene difluoride (PVDF) membranes (Immobilon, Millipore, Bedford, MA, USA). Blots were blocked with 5% nonfat dry milk in Tris-buffered saline (TBS: 20 mM Tris-HCl, 0.5 M NaCl, pH 7.5) and then incubated with primary antibodies overnight at 4 °C. Attached primary antibodies were identified using Alexa Fluor 680-linked anti-rabbit IgG (1:4000 dilution) (Molecular Probes, Eugene, OR, USA) and visualized using infrared detection with the LICOR Odyssey protein analysis system (LICOR, Lincoln, NE, USA). Antibodies to AQP2 phosphorylated at serine 269 (1:1000 dilution) were purchased from PhosphoSolutions, Aurora, CO, USA. Antibodies to AQP2 (1:2000 dilution) or UT-A1 (1:2000 dilution) were made in our laboratory [31,34]. Beta tubulin (1:5000 dilution) was used to determine the protein loading levels and was purchased from Abcepta, San Diego, CA, USA.

### 4.5. Phosphorylation

Metabolic labeling with [^32^P] orthophosphate was performed as previously described [35]. After 3 h of incubation of IM pieces in phosphate-free DMEM with 0.15 mCi/mL [^32^P] orthophosphate to radiolabel the ATP pool, 35 nM calyculin was added to the radiolabeling buffer for an additional 30 min. Following treatments, pieces were washed free of unincorporated ^32^P with phosphate-free DMEM, and ^32^P-labeled UT-A1 was immunoprecipitated as previously described [36]. Precipitated proteins were size separated by SDS-PAGE, and radiolabeled UT-A1 was determined by autoradiography of the dried gel. 

### 4.6. Statistics

All data are presented as mean ± SE. Data from tubule perfusion studies were analyzed using a paired Student’s *t*-test [37] if there were two measurement periods or a repeated-measure ANOVA if there were three periods. Each perfused tubule served as its own control, allowing a paired analysis of the data. Data from protein analysis studies with ADM were analyzed using a paired Student’s *t*-test. Data from protein analysis studies with calyculin were analyzed using a Student’s *t*-test. The criterion for statistical significance is *p* < 0.05.

## 5. Conclusions

The present study shows that calcineurin (PP2B) and PP2A regulate osmotic water permeability in rat IMCDs. Aldosterone acts through calcineurin to inhibit osmotic water reabsorption. ADM decreases osmotic water permeability by stimulating PP2A. Ser269 of AQP2 is a potential phosphorylation site that is regulated by PP2A. PP2A also reduces UT-A1 abundance, which reduces IM osmolality and the osmotic gradient for water reabsorption. This study provides direct functional evidence that phosphatases regulate osmotic water reabsorption. Future studies need to focus on investigating the effects of ADM-activated PP2A and aldosterone-stimulated calcineurin in water imbalance-related diseases such as hyponatremia associated with vasopressin escape, thus providing a new strategy for treating the disease.

## Data Availability

Not applicable.
